# Efeitos do Exercício em Choque Cardiogênico e Balão Intra-Aórtico: Um Relato de Caso

**DOI:** 10.36660/abc.20230537

**Published:** 2024-03-06

**Authors:** Vanessa M. Ferreira, Dayane Nunes Rodrigues, Carlos Alberto Mendez Contreras, João M. Rossi, Rui Fernando Ramos, Gustavo Oliveira, Mayron F. Oliveira

**Affiliations:** 1 Instituto Dante Pazzanese de Cardiologia Centro de Terapia Intensiva São Paulo SP Brasil Centro de Terapia Intensiva - Instituto Dante Pazzanese de Cardiologia São Paulo, SP - Brasil; 2 Instituto Dante Pazzanese de Cardiologia Unidade de Cirurgia Cardíaca São Paulo SP Brasil Unidade de Cirurgia Cardíaca - Instituto Dante Pazzanese de Cardiologia São Paulo, SP - Brasil; 3 Instituto Dante Pazzanese de Cardiologia Unidade de Insuficiência Cardíaca São Paulo SP Brasil Unidade de Insuficiência Cardíaca - Instituto Dante Pazzanese de Cardiologia São Paulo, SP - Brasil; 4 Unidade de Fisioterapia Grupo de Pesquisa VO_2_ Care São Paulo SP Brasil Grupo de Pesquisa VO_2_ Care - Unidade de Fisioterapia, São Paulo, SP - Brasil; 5 Grupo de Fisiologia do Exercício e Pesquisa Cardiopulmonar Integrada Lyon College Batesville AR EUA Grupo de Fisiologia do Exercício e Pesquisa Cardiopulmonar Integrada - EPIC Group, Exercise Science, Lyon College, Batesville, AR - EUA

**Keywords:** Choque cardiogênico, Balão Intra-aórtico, Exercício, Reabilitação Cardíaca, Fisioterapia

## Abstract

O presente relato de caso descreve o programa de exercícios aplicado a um paciente do sexo masculino, de 54 anos, internado com choque cardiogênico, aguardando transplante cardíaco e assistido por balão intra-aórtico, um dispositivo de suporte circulatório mecânico temporário.

O dispositivo de suporte circulatório mecânico temporário, um balão intra-aórtico, foi colocado na artéria subclávia esquerda, possibilitando o protocolo de exercícios. Antes e após um protocolo de exercícios, foram obtidos dados a partir de cateter de Swan-Ganz, amostra de sangue, peptídeo natriurético cerebral (NT-proBNP), proteína C reativa de alta sensibilidade (PCR-as), teste de caminhada de seis minutos (TC6min) e medição da saturação venosa de oxigênio (SvO_2_). O protocolo de treinamento físico envolveu a utilização de um cicloergômetro adaptado ao leito, sem carga, uma vez ao dia, por no máximo 30 minutos, até o limite da tolerância.

Não foram observados eventos adversos tampouco relacionados ao deslocamento do balão intra-aórtico durante o protocolo de exercícios. O programa de exercícios resultou em maior SvO_2_ com aumento do TC6min e menores escores de dispneia de Borg (312 metros vs. 488 metros e cinco pontos vs. três pontos, respectivamente). Após completar o protocolo de exercícios de dez dias, o paciente foi submetido a uma cirurgia de transplante cardíaco sem complicações e recuperação total na UTI.

O presente estudo demonstrou que o exercício é uma opção viável para pacientes com choque cardiogênico em uso de balão intra-aórtico e que é bem tolerado, além de não haver relatos de eventos adversos.

## Introdução

Diversos estudos indicaram treinamento físico como uma intervenção não farmacológica viável para pacientes com insuficiência cardíaca (IC). Entretanto, podem ocorrer episódios agudos de IC descompensada (ICD) e choque cardiogênico, levando à internação e longos períodos de repouso no leito.^1 - 3^

Recentemente, Reeves GR et al.^1^ demonstraram que a reabilitação iniciada durante a internação foi viável e bem tolerada em pacientes idosos internados com ICD. No entanto, o estudo não foi desenhado ou desenvolvido para avaliar definitivamente a eficácia ou segurança da intervenção de reabilitação física. Nessa linha, nosso grupo constatou que o exercício aeróbio pode beneficiar pacientes com ICD, com ou sem medicamentos invasivos, reduzindo o tempo de internação hospitalar e minimizando desfechos adversos.^4^

Contudo, para alguns pacientes gravemente doentes, dispositivos cardíacos de suporte circulatório mecânico temporários, como um balão intra-aórtico, podem ser necessários para melhorar o débito cardíaco e reduzir a mortalidade. Porém, tais dispositivos podem dificultar a realização da reabilitação cardíaca. Alguns artigos sobre pacientes estáveis com dispositivo de suporte circulatório mecânico temporário submetidos à reabilitação cardiovascular concluíram que o treinamento físico é seguro e recomendam a mobilização precoce, proporcionando excelente suporte para pacientes selecionados na forma de uma ponte para o transplante.^5 , 6^ Contudo, até o momento, nenhum estudo relatou a segurança e eficácia do exercício no choque cardiogênico com dispositivo de suporte circulatório mecânico temporário, como o balão intra-aórtico.

Portanto, o objetivo do nosso estudo foi apresentar um relato de caso examinando os efeitos do exercício no choque cardiogênico com balão intra-aórtico como dispositivo de suporte circulatório mecânico temporário.

## Relato de Caso

O presente relato de caso descreve a utilização de um programa de exercícios em um paciente do sexo masculino internado, de 54 anos, pesando 62 kg, com choque cardiogênico à espera de transplante cardíaco, que recebeu o suporte de um aparelho circulatório mecânico temporário, balão intra-aórtico.

O paciente apresentava histórico de tabagismo, alcoolismo e era portador de marca-passo, com fração de ejeção de 20% por ecodopplercardiograma e, de acordo com a *New York Hear Association* (NYHA), calssificado como classe IV D. Relatório prévio indicava função pulmonar normal com aumento da pressão de oclusão pulmonar. Assim que o paciente foi admitido na UTI, foi solicitado e instalado cateter de Swan-Ganz para acompanhamento do paciente durante todo o período de internação. O paciente recebeu inotrópico (Dobutamina 1,34 mcg/kg/min) com balão intra-aórtico inserido pela artéria subclávia esquerda, como um dispositivo de suporte circulatório mecânico temporário, sendo posteriormente indicado para transplante cardíaco. O estudo envolveu a análise de amostras de sangue e medição do peptídeo natriurético cerebral (NT-proBNP) e proteína C reativa de alta sensibilidade (PCR-as), além da realização de teste de caminhada de seis minutos (TC6min) e medição da saturação venosa de oxigênio (SvO_2_) antes e após o protocolo de exercícios. O paciente permaneceu com o balão intra-aórtico por 12 dias até o transplante cardíaco e o protocolo de exercícios foi realizado durante dez dias.

**Figura 1:**
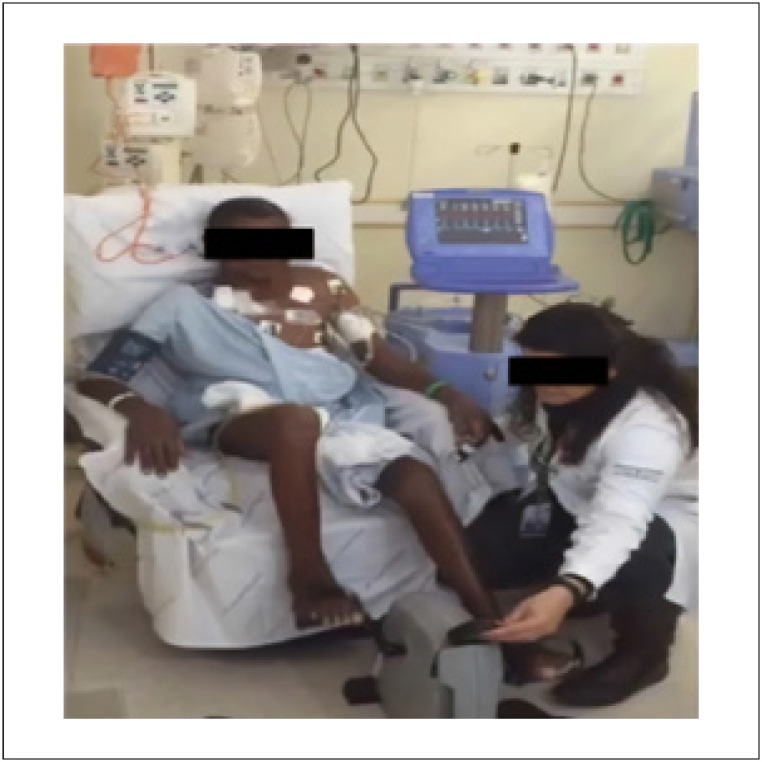
Protocolo de exercícios ilustrativo utilizando um dispositivo de suporte circulatório mecânico temporário, um balão intra-aórtico, via artéria subclávia.

Durante a internação, o protocolo de treinamento físico envolveu a utilização de um cicloergômetro adaptado ao leito, sem carga, uma vez ao dia, por no máximo 30 minutos, até o limite da tolerância ( Figura 1 ). Amostras hemodinâmicas e sanguíneas foram coletadas para mensuração da SvO_2_ antes e após o exercício (pré e pós). Além disso, ao final de cada sessão de exercício, o paciente foi solicitado a avaliar seu nível de "falta de ar" usando a escala de proporção de categorias de Borg.

O cateter de Swan-Ganz indicou redução do débito cardíaco em repouso (choque cardiogênico), conforme demonstrado nos dados basais ( Tabela 1 ). Com base no choque cardiogênico, a equipe médica decidiu utilizar balão intra-aórtico como dispositivo de suporte circulatório mecânico temporário durante o período de internação. O balão intra-aórtico foi inserido na artéria subclávia esquerda conforme discussão prévia entre médicos, cirurgiões e equipe de fisioterapia, possibilitando o protocolo de exercícios.^6 , 7^ Após avaliação criteriosa e liberação da equipe médica, o paciente iniciou os exercícios 48 horas após a colocação do balão intra-aórtico. A análise dos dados foi baseada em duas categorias - período basal e comparação entre pré- e pós-exercício. Os dados basais, coletados antes do início do protocolo de exercícios, representam os valores de repouso. Por outro lado, os dados hemodinâmicos apresentados na Tabela 1 , tanto pré quanto pós, representam os valores médios obtidos em dez sessões de exercícios realizadas ao longo dos dez dias de internação do paciente (valores médios registrados do dia 1 ao dia 10).

**Tabela 1:** Hemodinâmica e oxigenação tecidual em paciente com choque cardiogênico apoiado por dispositivo de suporte circulatório mecânico temporário - balão intra-aórtico

		Período Basal	Exercício	
			Pré	Pós
**Amostra sanguínea**				
	Hb, g/dL	12	-	-
	Plaquetas, mm^3^	107.000	-	-
	PCR-as, mg/L	4,8	-	-
	NT-proBNP, ρg/mL	48.100	-	-
	U, mg/dL	177	-	-
	Cr, mg/dL	3,6	-	-
	Leucograma, /mm^3^	5.200	-	-
	pH	7,39	7,40 ± 0,15	7,37 ± 0,20
	PvO_2_, mmHg	33,7	32,7 ± 2,3	39,2 ± 3,5
	PvCO_2_, mmHg	41,4	41,0 ± 1,2	41,1 ± 1,6
	HCO_3_^−^	25,2	25,3 ± 3,3	24,0 ± 3,1
	Lactato, mmol/L	2,2	2,1 ± 0,5	1,9 ± 0,4
**Swan-Ganz**				
	DC, l/min	3,1	3,0 ± 0,4	3,3 ± 0,7
	IC, l/min/m^2^	2,1	2,1 ± 0,3	2,4 ± 0,6
	VS, mL	33	32 ± 6	38 ± 7
	RVS, din•s/cm^5^	2.087	2.102 ± 389	1.912 ± 425
	RVP, din•s/cm^5^	509	505 ± 47	499 ± 41
	Pressão de oclusão pulmonar, mmHg	50	51 ± 4	51 ± 5
	DO_2_, mL/min	485	487 ± 55	490 ± 58
	⩒O_2_, mL/min	126	122 ± 23	131 ± 25
	SvO_2_, %	64	62 ± 6	67 ± 9

Hb: hemoglobina; PCR-as: proteína C reativa de alta sensibilidade; U: ureia; Cr: creatinina; BNP: peptídeo natriurético cerebral; DC: débito cardíaco; IC: índice cardíaco; VS: volume sistólico; RVS: resistência vascular sistêmica; RVP: resistência vascular pulmonar; DO_2_: fornecimento de oxigênio; ⩒O_2_: consumo de oxigênio; SvO_2_: saturação venosa de oxigênio; pH: potencial hidrogeniônico; PvO_2_: pressão venosa de oxigênio; PvCO_2_: pressão venosa de dióxido de carbono; HCO_3_-: bicarbonato; BE: excesso de base; SvO_2_: saturação de oxigênio do sangue venoso central.

É importante enfatizar que nenhum deslocamento do dispositivo foi observado durante ou após as sessões de treinamento. Não houve necessidade de nenhum procedimento de reposicionamento ou substituição e não foram observados eventos adversos relacionados ao balão intra-aórtico ao longo do estudo. Adicionalmente, antes de cada sessão do treinamento físico, tomamos precauções extras, verificando cuidadosamente o posicionamento e a estabilidade do balão intra-aórtico para garantir a segurança das sessões de exercícios. Estas verificações meticulosas foram realizadas para minimizar quaisquer possíveis riscos associados ao balão intra-aórtico durante os exercícios. Ademais, é importante ressaltar que as sessões de exercícios foram realizadas em cicloergômetro adaptado ao leito, e não envolveram caminhada. Esta decisão foi tomada com cautela para reduzir a probabilidade de deslocamento do dispositivo, uma vez que exercícios de caminhada ou resistidos podem representar um risco maior nesta população específica de pacientes.

Além disso, os valores relatados no exercício representam a média das medidas antes e após os blocos realizados ao longo do protocolo de exercícios ( Tabela 1 - valores pré e pós). O programa de exercícios resultou em maiores níveis de SvO_2_ com aumento do TC6min, além de menores escores de dispneia de Borg (312 metros vs. 488 metros e cinco pontos vs. três pontos, respectivamente). Após completar o protocolo de exercícios de 10 dias, o paciente foi submetido a uma cirurgia de transplante cardíaco sem complicações, tendo se recuperado na UTI e recebido alta sem quaisquer complicações.

## Discussão

Este é o primeiro relato de caso que examina o papel da reabilitação cardíaca no choque cardiogênico com dispositivo de suporte circulatório mecânico temporário, especificamente o balão intra-aórtico. Os resultados demonstraram que a reabilitação cardíaca não exacerbou os sintomas durante a internação e tampouco exigiu interrupção dos exercícios.

Recentemente, Chen et al.^8^ discutiram a segurança e a viabilidade de um protocolo de mobilização precoce para pacientes com balão intra-aórtico femoral como uma ponte para o transplante cardíaco. O estudo constatou que a mobilização precoce em pacientes selecionados com balão intra-aórtico femoral pode ser realizada com segurança e sucesso. As possíveis implicações dos achados de Chen et al.^8^ e de nosso relato de caso são que a mobilização precoce pode ser uma estratégia segura e eficaz para melhorar os resultados nesta população de pacientes e pode ajudar a melhorar os desfechos dos pacientes, reduzir a duração da internação e minimizar os custos com assistência médica. Entretanto, novas abordagens relacionadas a um dispositivo externo temporário poderiam ser colocadas na artéria subclávia, na tentativa de mobilizar precocemente os pacientes com ICD. Nessa linha, Macapagal et al.^9^ mostram que um paciente pré-transplante cardíaco com balão intra-aórtico inserido na artéria subclávia axilar de forma percutânea pode ser mobilizado com segurança. O estudo constatou que o balão intra-aórtico subclávio axilar permitiu que os pacientes fossem mobilizados com segurança enquanto aguardavam o transplante, com os pacientes sendo mobilizados 1,39 (±1,41) dia após a inserção, em vez de três dias como no estudo de Chen et al.^8^ Nossos resultados corroboram os de Macapagal et al.^9^ e demonstram que o balão aórtico inserido na artéria subclávia é viável e proporciona uma mobilização muito precoce, evitando, assim, as complicações do repouso prolongado no leito, em comparação com o repouso absoluto no leito para pacientes que possuem um balão intra-aórtico femoral tradicional. Nossos resultados nos permitem aprofundar esse assunto, uma vez que a capacidade de mobilizar pacientes com choque cardiogênico com um dispositivo externo temporário pode melhorar os resultados e a qualidade de vida dos pacientes, reduzindo o risco de complicações associadas ao repouso prolongado no leito, como trombose venosa profunda, embolia pulmonar e atrofia muscular.

Estudos relataram perda de massa muscular durante a internação foi independentemente associada à um maior risco de mortalidade tardia em pacientes com IC após internação aguda.^2^ Estudo recente sugere que aumentos no índice de massa corporal e melhor massa muscular esquelética podem fornecer proteção contra mortalidade por todas as causas em pacientes com IC após alta hospitalar causada por ICD.^2^ Além disso, Lopez et al.^2^ e Hasin et al.^10^ sugeriram que pacientes com menor massa muscular após um período de internação ou aqueles com menor capacidade de locomoção apresentavam maior taxa de mortalidade em comparação aos seus pares. Recentemente, Oliveira et al.^4^ demonstraram que a realização de exercícios durante a internação pode reduzir o tempo de internação de pacientes com ICD sem causar complicações relacionadas ao exercício. No presente relato de caso, observamos um padrão semelhante, o que sugere que pode ter ocorrido uma melhora na distância percorrida no TC6min e possivelmente no metabolismo muscular intrínseco, incluindo melhorias na função endotelial.^11 , 12^ Além disso, vale ressaltar que o TC6min e a dispneia têm sido associados a taxas de mortalidade e reinternação em pacientes com IC.^13 , 14^ O aumento na distância total percorrida com redução da dispneia (Borg) durante um protocolo de exercícios pode, portanto, reduzir a probabilidade de eventos adversos em pacientes internados em uso de dispositivo de suporte circulatório mecânico temporário.

Ademais, estudo anterior constatou que valores baixos de SvO_2_ estavam associados a maiores taxas de mortalidade em UTI.^15^ Nosso estudo indica que os níveis de SvO_2_ estavam reduzidos, sugerindo que mecanismos musculares intrínsecos poderiam desempenhar um papel na tolerância ao exercício em pacientes com choque cardiogênico. No entanto, os programas de exercícios melhoraram os níveis de SvO_2_, o que pode indicar uma utilização mais eficiente do oxigênio fornecido nos músculos em exercício. Vale ressaltar que medidas diretas obtidas por meio do cateter de Swan-Ganz indicaram medidas cardíacas estáveis ou discretamente melhores, sugerindo que o exercício não interferiu na hemodinâmica nem aumentou o risco ao paciente durante o exercício. É possível supor que tanto o exercício quanto o dispositivo de suporte circulatório mecânico temporário podem levar à melhora da função pulmonar durante o exercício, reduzindo a incompatibilidade ventilação/perfusão.^12^ Em pacientes com IC/choque cardiogênico, as adaptações pulmonares relacionadas ao exercício são importantes e o suporte temporário do dispositivo de suporte circulatório mecânico pode normalizar as pressões da artéria pulmonar, possivelmente reduzindo as limitações do exercício. No presente relato de caso, observamos discreta melhora no débito cardíaco durante o exercício, sem qualquer aumento na pressão de oclusão pulmonar. Nossos achados indicam a possibilidade de tais ocorrências, pois todos esses fatores juntos nos levam a especular que descarregar o sistema cardiovascular e permitir os benefícios do exercício podem ter contribuído para a melhora da função periférica (SvO_2_, muscular e/ou endotelial).

Gostaríamos de enfatizar que este é um relato de caso e, como tal, nossos achados devem ser analisados com cautela. É importante ressaltar que o protocolo de exercícios foi realizado durante um período de apenas dez dias e não medimos sarcopenia e/ou força muscular. No entanto, obtivemos dados indiretos relacionados à musculatura (aumento do TC6min com redução da dispneia e alterações nos valores de SvO_2_) que poderiam nos levar a sugerir tais adaptações. Além disso, como este foi o primeiro estudo a investigar o uso de um dispositivo de suporte circulatório mecânico temporário em pacientes com choque cardiogênico durante o exercício, a duração limitada foi apropriada. Entretanto, não há diretrizes quanto à melhor abordagem para realização de exercícios ou prescrição de exercícios para pacientes com dispositivo de suporte circulatório mecânico temporário ou em choque cardiogênico.

Tanto quanto é de nosso conhecimento, este foi o primeiro relato de caso de exercício em paciente com choque cardiogênico. Nessa linha, decidimos seguir nosso protocolo anterior de exercícios para ICD.^4^ Porém, são necessários estudos adicionais para determinar a prescrição do exercício, o momento ideal e a duração da mobilização precoce, bem como os possíveis benefícios em outras populações de pacientes submetidos a procedimentos de transplante cardíaco. Vale ressaltar que os dados cirúrgicos foram incertos devido à rara condição pré-transplante, que poderia ter surgido em qualquer momento do estudo. Ademais, não tivemos um grupo controle e estudos maiores realizados no futuro devem considerar um grupo controle para melhor concretizar e confirmar nossos achados.

Concluindo, o presente estudo demonstrou que o exercício é uma opção viável para pacientes em choque cardiogênico em uso de balão intra-aórtico como dispositivo de suporte circulatório mecânico temporário e que é bem tolerado, sem relatos de eventos adversos.
