# Supplementary material for: Efeitos do Exercício em Choque Cardiogênico e Balão Intra-Aórtico: Um Relato de Caso
Source: Arq Bras Cardiol. 2024 Mar 6;121(1):e20230537. [Article in Portuguese] doi: 10.36660/abc.20230537 (PMC11081090; doi:10.36660/abc.20230537)
Supplement: Translation

# Translation: The Effects of Exercise on Cardiogenic Shock with an Intra-Aortic Balloon Pump: A Case Report

**Authors:** Vanessa M. Ferreira, Dayane Nunes Rodrigues, Carlos Alberto Mendez Contreras, João M. Rossi, Rui Fernando Ramos, Gustavo Oliveira, Mayron F. Oliveira

**Affiliations:** 1 São Paulo SP Brazil Centro de Terapia Intensiva - Instituto Dante Pazzanese de Cardiologia, São Paulo, SP - Brazil; 2 São Paulo SP Brazil Unidade de Cirurgia Cardíaca - Instituto Dante Pazzanese de Cardiologia, São Paulo, SP - Brazil; 3 São Paulo SP Brazil Unidade de Insuficiência Cardíaca - Instituto Dante Pazzanese de Cardiologia, São Paulo, SP - Brazil; 4 São Paulo SP Brazil Grupo de Pesquisa VO_2_Care - Unidade de Fisioterapia, São Paulo, SP - Brazil; 5 Batesville AR USA Exercise Physiology and Integrated Cardiopulmonary Research Group - EPIC Group, Exercise Science, Lyon College, Batesville, AR - USA

**Keywords:** Cardiogenic Shock, Intra-Aortic Balloon Pumping, Exercise, Cardiac Rehabilitation, Physical Therapy Modalities

## Abstract

This case report describes the exercise program on a hospitalized 54-year-old male patient with cardiogenic shock waiting for a heart transplant assisted by an intra-aortic balloon pump, a temporary mechanical circulatory support device.

The temporary mechanical circulatory support device, an intra-aortic balloon pump, was placed in the left subclavian artery, enabling the exercise protocol. Measurements and values from Swan-Ganz catheter, blood sample, brain natriuretic peptide (NT-proBNP), and high-sensitivity C-reactive protein (hs-CRP), as well as the six-minute walk test (6MWT) and venous oxygen saturation (SvO_2_) were obtained before and after an exercise protocol. The exercise training protocol involved the use of an unloaded bed cycle ergometer once a day, for a maximum of 30 minutes, to the tolerance limit.

No adverse events or events related to the dislocation of the intra-aortic balloon pump were observed during the exercise protocol. The exercise program resulted in higher SvO_2_levels, with an increased 6MWT with lower Borg dyspnea scores (312 meters vs. 488 meters and five points vs. three points, respectively). After completing the ten-day exercise protocol, the patient underwent a non-complicated heart transplant surgery and a full recovery in the ICU.

This study showed that exercise is a feasible option for patients with cardiogenic shock who are using an intra-aortic balloon pump and that it is well-tolerated with no reported adverse events.

## Introduction

Several studies have indicated that exercising can serve as a viable non-pharmacological intervention for patients with heart failure (HF). However, acute decompensated HF (ADHF) and cardiogenic shock episodes may occur, leading to hospitalization and extended periods of bed rest.^1 - 3^

Recently, Reeves GR et al.^1^found that a physical rehabilitation intervention initiated during hospitalization was feasible and well-tolerated in older patients hospitalized with ADHF. However, the study was not designed or powered to definitively assess the efficacy or safety of the physical rehabilitation intervention. In this line, our group found that aerobic exercise can benefit patients with ADHF, with or without invasive medications, by reducing the length of hospital stay and minimizing adverse outcomes.^4^

However, for some severely ill patients, temporary mechanical circulatory support cardiac devices like an intra-aortic balloon pump may be necessary to improve cardiac output and reduce mortality, but such devices may make it difficult to conduct cardiac rehabilitation. Some papers regarding stable patients with cardiac temporary mechanical circulatory support devices undergoing cardiovascular rehabilitation concluded that exercising is safe and recommended early mobilization, providing excellent support for selected patients as a bridge to transplant.^5 , 6^Still, to date, no studies have reported on the safety and efficacy of exercising in cardiogenic shock that has a temporary mechanical circulatory support device, such as an intra-aortic balloon pump.

Therefore, our study aims to present a case report examining the effects of exercise on cardiogenic shock with an intra-aortic balloon pump as a temporary mechanical circulatory support device.

## Case Report

This case report describes the use of an exercise program on a hospitalized 54-year-old male patient (weight - 62 kg) with cardiogenic shock waiting for a heart transplant, who was assisted with a temporary mechanical circulatory support device, an intra-aortic balloon pump.

The patient had a history of smoking and alcohol use and had a pacemaker, with an ejection fraction of 20% by Doppler echocardiography, being classified as New York Heart Association (NYHA) IV class D. A previous report indicated that the patient exhibited normal lung function with a higher pulmonary wedge pressure. As soon as the patient was admitted to ICU, a Swan-Ganz catheter was requested and installed to monitor the patient's evaluation throughout the hospitalization period. The patient received an inotropic agent (Dobutamine 1.34 mcg/kg/min) with an intra-aortic balloon pump inserted through the left subclavian artery as a temporary mechanical circulatory support device, and the patient was subsequently listed for a heart transplant. The study involved measuring blood samples, brain natriuretic peptide (NT-proBNP), and high-sensitivity C-reactive protein (hs-CRP), as well as conducting a six-minute walk test (6MWT) and venous oxygen saturation (SvO_2_) before and after an exercise protocol. The patient had the intra-aortic balloon pump in place for 12 days until the heart transplant, and the exercise protocol was performed for ten days.

The exercise training protocol involved the use of an unloaded bed cycle ergometer once a day, for a maximum of 30 minutes, to the tolerance limit, during the hospitalization period ( Figure 1 ). Hemodynamic and blood samples were taken to measure SvO_2_before and after the exercise (pre and post). Additionally, at the end of each exercise session, the patient was asked to rate their level of "shortness of breath" using the Borg Category-Ratio scale.

**Figure 1:** An illustrative exercise protocol using a temporary mechanical circulatory support device, an intra-aortic balloon pump, via the subclavian artery.

The Swan-Ganz catheter indicated reduced cardiac output at rest (cardiogenic shock), as shown in baseline data ( Table 1 ). Based on cardiogenic shock, the medical team decided to use an intra-aortic balloon pump as a temporary mechanical circulatory support device during his hospitalization. The intra-aortic balloon pump was placed in the left subclavian artery as per the previous discussion by the clinicians, surgeons, and physiotherapy team, enabling the exercise protocol.^6 , 7^After a careful evaluation and clearance from the medical team, the patient commenced exercising 48 hours following the placement of the intra-aortic balloon pump. Data analyses were based on two categories - baseline and the comparison between pre- and post-exercise. The baseline data, collected before the initiation of the exercise protocol, represents the resting values. On the other hand, the hemodynamic data presented in Table 1 , both pre and post, represents the average values obtained from ten exercise sessions conducted over the ten days of the patient's hospitalization (mean values recorded from day one to day ten).

**Table 1:** Hemodynamics and tissue oxygenation in cardiogenic shock patient supported by a temporary mechanical circulatory support device - intra-aortic balloon pump

		Baseline	Exercise	
			Pre	Post
**Blood-sample**				
	Hb, g/dL	12	-	-
	Platelets, mm^3^	107,000	-	-
	hs- RCP, mg/L	4.8	-	-
	NT-proBNP, ρg/mL	48,100	-	-
	U, mg/dL	177	-	-
	Cr, mg/dL	3.6	-	-
	Leucogram, /mm^3^	5,200	-	-
	pH	7.39	7.40 ± 0.15	7.37 ± 0.20
	PvO_2_, mmHg	33.7	32.7 ± 2.3	39.2 ± 3.5
	PvCO_2_, mmHg	41.4	41.0 ± 1.2	41.1 ± 1.6
	HCO_3_^−^	25.2	25.3 ± 3.3	24.0 ± 3.1
	Lactate, mmol/L	2.2	2.1 ± 0.5	1.9 ± 0.4
**Swan-Ganz**				
	CO, l/min	3.1	3.0 ± 0.4	3.3 ± 0.7
	CI, l/min/m^2^	2.1	2.1 ± 0.3	2.4 ± 0.6
	SV, mL	33	32 ± 6	38 ± 7
	SVR, dyn•s/cm^5^	2,087	2,102 ± 389	1,912 ± 425
	PVR, dyn•s/cm^5^	509	505 ± 47	499 ± 41
	Pulmonary wedge pressure, mmHg	50	51 ± 4	51 ± 5
	DO_2_, mL/min	485	487 ± 55	490 ± 58
	⩒O_2_, mL/min	126	122 ± 23	131 ± 25
	SvO_2_, %	64	62 ± 6	67 ± 9

Hb: hemoglobin; hs-CRP: high sensitive C-reactive protein; U: Urea; Cr: Creatinine; BNP: brain natriuretic peptide; CO: cardiac output; CI: cardiac index; SV: stroke volume; SVR: systemic vascular resistance; PVR: pulmonary vascular resistance; DO_2_: oxygen delivery; ⩒O_2_: oxygen consumption; SvO_2_: Venous oxygen saturation; pH: hydrogen potential; PvO_2_: venous oxygen pressure; PvCO_2_: venous carbon dioxide pressure; HCO_3_-: bicarbonate; BE: base excess; SvO_2_: oxygen saturation of central venous blood.

We want to emphasize that no device displacement was observed during or following the training sessions. There was no need for any repositioning procedures or replacements, and no adverse events related to the intra-aortic balloon pump were observed throughout the study. Additionally, before each exercise training section, we took extra precautions by carefully double-checking the placement and stability of the intra-aortic balloon pump to ensure the safety of the exercise sessions. These meticulous checks were conducted to minimize any potential risks associated with the intra-aortic balloon pump during exercise. Furthermore, it is important to note that the exercise sessions were performed using a bed cycle ergometer rather than walking. This decision was made with careful consideration to reduce the likelihood of device displacement, as walking or weight-bearing exercises might carry a higher risk in this particular patient population.

In addition, the values reported in the exercise represent the average of pre- and post-section measurements taken over the exercise protocol ( Table 1 - pre- and post-values). The exercise program resulted in higher SvO_2_levels with increased 6MWT results, in addition to lower Borg dyspnea scores (312 meters vs. 488 meters and five points vs. three points, respectively). After completing the ten-day exercise program, the patient underwent a non-complicated heart transplant surgery and a full recovery in the ICU, being discharged without any complications.

## Discussion

This is the first case report that examines the role of cardiac rehabilitation in cardiogenic shock with a temporary mechanical circulatory support device, specifically the intra-aortic balloon pump. The results showed that cardiac rehabilitation did not exacerbate symptoms during hospitalization or necessitate interruption of the exercises.

Recently Chen et al.^8^discussed the safety and feasibility of an early mobilization protocol for patients with femoral intra-aortic balloon pumps as a bridge to heart transplant. The study found that early mobilization in select patients with femoral intra-aortic balloon pumps can be performed safely and successfully. The potential implications of the Chen et al.^8^findings and our case report are that early mobilization can be a safe and effective strategy to improve outcomes in this patient population, and it may help to improve patient outcomes, shorten hospital stays, and reduce healthcare costs. However, new approaches related to a temporary external device could be placed on the subclavian artery, in an attempt to early mobilize ADHF patients. In this line, Macapagal et al.^9^shows that a pre-heart transplant patient with a percutaneously placed axillary subclavian intra-aortic balloon pump can be safely mobilized. The study found that the axillary subclavian intra-aortic balloon enabled patients to be safely mobilized while awaiting transplant, with patients being mobilized at 1.39 (±1.41) days after insertion instead of three days as mentioned in the Chen et al.^8^study. Our results agree with Macapagal et al.^9^and show that the subclavian aortic-balloon pump is feasible and allows very early mobilization, thereby avoiding the complications of prolonged bed rest, as compared to complete bed rest for patients who have a traditional femoral intra-aortic balloon pump. Our results allow us to go deeper into this subject, since the ability to mobilize patients with cardiogenic shock with a temporary external device might improve patient outcomes and quality of life by reducing the risk of complications associated with prolonged bed rest, such as deep vein thrombosis, pulmonary embolism, and muscle atrophy.

Studies have reported a loss of muscle mass during hospitalization, which has been found to be independently associated with a higher risk of late mortality in patients with HF following acute hospitalization.^2^A recent study suggests that increases in body mass index and better skeletal muscle mass may protect against all-cause mortality in patients with HF after hospital discharge caused by ADHF.^2^Additionally, Lopez et al.^2^and Hasin et al.^10^suggested that patients with lower muscle mass after a hospitalization period or those with lower walking capacity had higher mortality rates compared to their counterparts. Recently, Oliveira et al.^4^demonstrated that performing exercise during hospitalization can reduce the length of stay for patients with ADHF without causing any exercise-related complications. In this case report, we observed a similar pattern, which suggests an enhancement in walking distance by 6MWT and, possibly, intrinsic muscle metabolism, including possible improvements in endothelial function.^11 , 12^Furthermore, it is worth noting that the 6MWT and dyspnea have been linked to mortality and re-hospitalization rates in patients with HF.^13 , 14^An increase in the total distance covered with a reduced Borg dyspnea during an exercise protocol may, therefore, decrease the likelihood of adverse events in hospitalized patients using temporary mechanical circulatory support devices.

Furthermore, a prior study found that lower values of SvO_2_were associated with higher mortality rates in ICU.^15^Our study indicates that SvO_2_levels were reduced, suggesting that intrinsic muscle mechanisms could play a role in exercise tolerance among patients with cardiogenic shock. However, exercise programs improved SvO_2_levels, which might indicate a more efficient utilization of oxygen delivery in exercising muscles. It is worth noting that direct measurements obtained through a Swan-Ganz catheter indicated stable or slightly improved cardiac measurements, suggesting that exercise did not interfere with hemodynamics or increase the risk to the patient during exercise. We could assume that both exercise and the temporary mechanical circulatory support device may lead to improved pulmonary function during exercise by reducing ventilation/perfusion mismatching.^12^In HF patients/cardiogenic shock, exercise-related pulmonary adaptations are important, and temporary mechanical circulatory support devices can normalize pulmonary artery pressures, potentially improving exercise limitations. In this case report, we observed slight enhancements in cardiac output during exercise without any increase in pulmonary wedge pressure. Our findings indicate the possibility of such occurrences, as all these factors together lead us to speculate that unloading the cardiovascular system and providing exercise benefits may have contributed to improved peripheral function (SvO_2_, muscle, and/or endothelial).

We want to emphasize that this is a case report and, as such, our findings should be analyzed with caution. It is important to note that the exercise protocol was conducted over only ten days and we did not measure sarcopenia and/or muscle strength. Nonetheless, we had indirect muscle-related data (increase in 6MWT with reduced dyspnea and changes in SvO_2_values) that could lead us to suggest such adaptations. Additionally, since this was the first study to investigate the use of a temporary mechanical circulatory support device in cardiogenic shock patients during exercise, the limited duration was appropriate. However, there are no guidelines regarding the best approach toward exercise prescription or methodology for patients with temporary mechanical circulatory support devices or in cardiogenic shock.

To the best of our knowledge, this was the first case report of exercise in a cardiogenic shock patient. In this line, we decided to follow our previous ADHF exercise protocol.^4^However, further studies are needed to determine the exercise prescription, optimal timing, and duration of early mobilization, as well as the potential benefits in other patient populations undergoing heart transplant procedures. Additionally, it is worth noting that surgical data was uncertain due to the rare pre-transplant condition, which could have arisen at any point during the study. Furthermore, we did not have a control group. Therefore, larger future studies should consider a control group to better accomplish and confirm our findings.

In conclusion, this study has shown that exercising is a feasible option for patients with cardiogenic shock who are using an intra-aortic balloon pump as a temporary mechanical circulatory support device and that it is well tolerated with no reported adverse events.
